# Antitumor Mechanisms of *Lycium barbarum* Fruit: An Overview of In Vitro and In Vivo Potential

**DOI:** 10.3390/life14030420

**Published:** 2024-03-21

**Authors:** Maria Rosaria Miranda, Vincenzo Vestuto, Giuseppina Amodio, Michele Manfra, Giacomo Pepe, Pietro Campiglia

**Affiliations:** 1 Department of Pharmacy, University of Salerno, Via G. Paolo II, Fisciano, 84084 Salerno, Italy; mmiranda@unisa.it (M.R.M.); gipepe@unisa.it (G.P.); pcampiglia@unisa.it (P.C.); 2 PhD Program in Drug Discovery and Development, University of Salerno, Fisciano, 84084 Salerno, Italy; 3 Department of Medicine, Surgery and Dentistry “Scuola Medica Salernitana”, University of Salerno, 84084 Salerno, Italy; gamodio@unisa.it; 4 Department of Science, University of Basilicata, Viale Dell’Ateneo Lucano 10, 85100 Potenza, Italy; michele.manfra@unibas.it; 5 National Biodiversity Future Center (NBFC), 90133 Palermo, Italy

**Keywords:** *Lycium barbarum*, goji berries, antioxidants, pro-oxidants, breast cancer, doxorubicin, immune cells stimulation

## Abstract

*Lycium barbarum*, known as goji berry or wolfberry, is a fruit long associated with health benefits, showing a plethora of effects ranging from antioxidant, anticancer, anti-inflammatory, and immunomodulatory effects. Its potential is attributed to the significant presence of polysaccharides, glycopeptides, polyphenols, flavonoids, carotenoids, and their derivatives. These compounds effectively counteract the action of free radicals, positively influencing cellular balance and intracellular signaling, contributing to overall cell health and function acting on multiple molecular pathways. Several fractions extracted from goji berries demonstrate antitumor properties, particularly effective against breast cancer, without showing cytotoxic effects on normal human cells. Hence, the review explored the fundamental traits of bioactive elements in *Lycium barbarum* and their potential in cancer treatment and, specifically, breast cancer. It focused on elucidating wolfberry’s influenced biochemical pathways, its synergism with anticancer drugs, and its potential to alleviate the side effects associated with existing cancer treatments.

## 1. Introduction

Since ancient times, humanity has turned to natural substances from plants for the formulation of medicinal remedies aimed at alleviating and curing diseases [1,2,3,4].

To date, the prevalence of natural therapy is on the rise, supported by evidence that numerous molecules, including polyphenols, flavonoids, peptides, vitamins, and minerals, typically found in plant matrices, exhibit antioxidant, anti-inflammatory, and immunostimulant properties. These substances can play a key role in the prevention and possibly in the therapy of chronic conditions, including cardiovascular diseases, neurodegenerative disorders, gastrointestinal issues, liver diseases, diabetes, and certain types of cancer [5,6,7,8,9,10,11].

Some examples include glycyrrhizin, which is employed in the treatment of viral hepatitis and herpes simplex virus (HSV) by interfering with the intracellular transport of viral antigens [12,13]. Capsaicin, deployed as a topical analgesic, selectively activates TRPV1, a Ca^2+^-permeable cationic ion channel that is enriched in the terminals of certain nociceptors [14]. Curcumin is beneficial for inflammatory pathologies, such as arthritis and cardiovascular disorders, by inhibiting mitogen-activated protein kinase (MAPK) family and nuclear factor κB (Nf-κB) signal pathways [15].

Catechins can be PPAR-γ agonists, in addition to targeting nuclear factor erythroid 2–related factor 2 (Nrf2) and unfolded protein response (UPR) target genes to exert their potent antioxidant effect that hunts free radicals by chelating metal ions to form an inactive complex. They play a beneficial role in supporting heart health by preserving the structure of cardiac muscle cells and reducing the risk of conditions like atherosclerosis and cardiac hypertrophy as well as neurodegenerative disorders [16,17]. Onions and scallions are increasingly being recognized as potential nutraceuticals due to their rich content of flavonoids and sulfur-containing compounds like thiosulfinates and allyl sulfides, which impart various health-promoting properties stimulating the production of detoxification enzymes, such as glutathione S-transferases (GSTs), contributing to support the liver’s scavenging mechanisms [18,19,20].

Additionally, nutraceuticals such as *Spirulina platensis*, *Ganoderma lucidum*, and *Moringa oleifera* are more and more known for their anti-inflammatory properties through blocking the NLR family pyrin domain containing 3 (NLRP3) inflammasome, reducing the activation of pro-inflammatory cytokines. These supplements are increasingly used alongside conventional cancer treatments to enhance the well-being and quality of life for patients [21,22].

Menthol, a natural compound found in mint plants such as peppermint and spearmint, acts as an agonist of the Transient Receptor Potential Melastatin 8 (TRPM8) channel. TRPM8 is a cold- and menthol-sensitive receptor expressed in sensory neurons, particularly those involved in temperature sensation and pain perception as well as prostate cancer. Menthol induces a cooling sensation followed by analgesia or pain relief [23,24].

Research has shown that melanoidins extracted from coffee silver skin and other sources exhibit antioxidant activity and may provide some level of protection against UV-induced skin damage. Melanoidins are complex, high molecular weight compounds formed during the Maillard reaction, which occurs between amino acids and reducing sugars during thermal processing, such as roasting and baking. Melanoidins contribute to the characteristic color, aroma, and flavor of many foods, including coffee, bread, beer, and chocolate. However, the exact mechanisms by which melanoidins exert their photoprotective effects are not fully understood [25] (Table 1).

However, despite the presence of numerous studies in the scientific literature on natural products, the understanding of the activity of such substances is only partial. For many of these substances, there remains a lack of comprehensive understanding regarding the specific components responsible for their purported biological effects and the mechanisms through which they operate. This is largely due to the inherent complexity and variability found within natural matrices. Despite this, given that many diseases are not influenced by a single molecular target but frequently arise from multifactorial causes, numerous studies have demonstrated that disease resistance is less likely when facing a combination of compounds compared to individual active constituents. This is among the factors driving the increasing trend of combining natural products with pharmacological therapies. Such an approach aims to augment treatment efficacy while mitigating potential side effects [26,27,28,29,30].

Among the wide range of plant-derived compounds that are garnering significant interest for their bioactive composition, exotic berry-type fruits stand out. In particular, various studies, conducted both in vitro and in vivo, have highlighted the impressive biological capabilities of *L. barbarum*, better known as goji berries [31,32,33,34,35,36].

*L. barbarum* belongs to the group of plants that show numerous beneficial effects thanks to the presence of high levels of bioactive compounds. Fractions extracted from goji berries, depending on their types and the abundance of individual compounds, show antiproliferative properties against various types of cancer, including breast cancer [37,38].

This last property is particularly interesting because, despite the progress of recent years in the treatment of breast cancer, it remains a worldwide problem associated with high mortality rates. Furthermore, the mechanism of action of goji berries on breast cancer is not fully elucidated, but several potential pathways have been proposed from research findings based on in vitro and animal studies.

Below, we provide an overview of different breast cancer types, emphasizing their key features to establish connections with various commercially available breast cancer cell lines. This approach aims to deepen comprehension in subsequent sections, particularly in investigating the mechanisms of *Lycium barbarum* against various breast cancer cells in both in vitro and in vivo experiments documented in existing literature.

Breast cancers can be classified at a molecular level based on various characteristics, including gene expression patterns and specific molecular markers. Some key molecular classifications of breast cancer include the following:

1. **Hormone Receptor Status:** Breast cancers can be categorized based on the presence or absence of hormone receptors: estrogen receptor (ER) and progesterone receptor (PR). Luminal A represents some subtypes of hormone receptor-positive (ER+ and/or PR+), HER2-negative (HER2-) breast cancer, and typically has low levels of the protein Ki-67, indicating slower cell proliferation. It often has a better prognosis and tends to respond well to hormone-based therapies. ZR-75-1, T-47D, MCF-7, and MDA-MB-415 all represent this subtype [39].
2. Luminal B subtypes are also hormone receptor-positive (ER+ and/or PR+), but they might have higher levels of Ki-67, indicating faster cell growth. Some Luminal B tumors may also express HER2 (Luminal B/HER2+), influencing treatment decisions. MDA-MB-330 and ZR-75-30 are often the breast cancer cell lines of choice to represent the Luminal B subtype [39].
3. **HER2 Status:** Human Epidermal Growth Factor Receptor 2 (HER2) is a protein that can promote the growth and division of cancer cells. Breast cancers can be classified as HER2-positive (HER2+) if they overexpress this protein, which can influence treatment decisions. MDA-MB-453, HCC1569, SUM190PT, AU565, and SK-BR-3 represent HER2-enriched breast cancer cell lines that do not have hormonal receptors for ER or PR [39].
4. **Basal-like/Triple-Negative Breast Cancer (TNBC):** This subtype lacks the expression of ER, PR, and HER2 receptors characterized by its gene expression profile, resembling that of basal cells in the breast. A significant portion of TNBC tumors can be classified as basal-like and tend to be the most aggressive presenting fewer targeted treatment options. BT-549, BT-20, CAL148, MDA-MB-157, and MDA-MB-231 are all breast cancer cell lines that have been classified as TNBC [39].
5. **Claudin-low:** These tumors are a subset of the basal-like subtype and are characterized by the low expression of tight junction proteins known as claudins. They have a poorer prognosis since the presence of claudin-low tumors has been associated with higher aggressiveness, exhibiting features associated with epithelial-to-mesenchymal transition (EMT). MDA-MB-157, BT-549, and MDA-MB-231 are used to study claudin-low breast cancer [40].
6. **Molecular Subtypes (e.g., PAM50), genomic instability and mutations:** Molecular profiling using gene expression assays like PAM50 identifies distinct subtypes such as Luminal A, Luminal B, HER2-enriched, and basal-like. These subtypes have different gene expression patterns and respond differently to treatments. Moreover, some classifications consider genomic instability and specific genetic mutations like BRCA1 and BRCA2 mutations, which can predispose individuals to breast cancer since these human genes produce proteins responsible for suppressing tumors and play a crucial role in repairing damaged DNA. Certainly, all these genetic variations are specific to each cell line.

These molecular classifications help guide treatment strategies and prognostic assessments, enabling more personalized and targeted approaches to managing breast cancer [41,42,43].

Hence, the goal of this review is to present a thorough examination of the anticancer properties of bioactive compounds found in goji berries, focusing specifically on their impact on breast cancer both independently and when co-administered with anticancer drugs used in clinical practice.

## 2. *Lycium barbarum*

Goji berries are the fruit of *L. barbarum*, which is native to regions such as China, Mongolia, and the Himalayas, representing one of the most widespread members of the *Solanaceae* family.

There are three species of *Lycium*, including *L. chinense*, *L. ruthenicum*, and *L. barbarum.* This latter is the most widespread in China and has been widely used in traditional Chinese medicine (TCM) for about 2300 years [31,44].

Furthermore, only the fruit of *L. barbarum* and the root bark of both *L. barbarum* and *L. chinense* are recorded in the Chinese Pharmacopoeia. Although *L. ruthenicum* is commonly used in traditional Tibetan medicine, it has gained increasing global attention [45,46].

*L. barbarum* and *L. chinense* have nearly identical levels of bioactive substances, with only minor differences in the quantity of components. For instance, the content of derivatives of chlorogenic acid and rutin is notably higher in *L. chinense* compared to *L. barbarum.*

*L. ruthenicum* presents more differences, including morphological features such as its black berries. Moreover, anthocyanins, a type of water-soluble natural pigment, are found exclusively in the fruits of *L. ruthenicum*. Pigments extracted from these berries are widely used as natural food colorants.

To uncover the relationship between *Lycium* species, molecular techniques such as SSR (simple sequence repeat) genotyping, chloroplast genome, single nucleotide polymorphism, gene cloning, etc., have been employed. These phylogenetic analyses of the three *Lycium* species have indeed revealed a closer relationship between *L. barbarum* and *L. chinense*, in comparison to *L. ruthenicum* [47,48,49].

The berry, leaf, root, and shoot of *L. barbarum* are still used as a food and/or local medicine and are even known as a “superfood” in Europe and North America. The mature berry, elliptical in shape with a bright red-orange color, measures approximately 1–2 cm in length and contains 20–40 small seeds. The ripe fruit is esteemed for its sweet taste and spicy aroma, appreciated for its health-promoting properties as well as its organoleptic qualities [32,50,51,52].

More than 200 different components, including carotenoids, phenylpropanoids, flavonoids, polyphenols, and polysaccharides have emerged from various characterization studies of goji berries extracts. Numerous capacities are attributed to these bioactive substances, including antiaging, antioxidant, antidiabetic, anticancer, cytoprotective, antimicrobial, neuroprotective, and immunomodulatory properties [45,53,54,55].

## 3. Bioactive Composition of *L. barbarum*

The bioactivity of plants is subject to numerous factors including variety, ripening stage, geographical origin, and prevailing climatic conditions, all of which significantly affect their chemical composition [56,57,58]. According to the scientific literature, the main classes of bioactive molecules associated with the biological activities of goji berries are polyphenols and polysaccharides. In recent years, alkaloids, fatty acids, carotenoids, and tocopherols have also been reported for their bioactivities [59].

In the upcoming paragraphs, our focus will specifically delve into the components found in *L. barbarum* fruits linked to their anticancer properties.

### 3.1. Polyphenolic Compounds

Phenolic compounds are abundant secondary metabolites in the berries, leaves, and roots of *L. barbarum* [60,61]. Around 88 distinct phenolic compounds have been recognized, with flavonoids constituting the predominant category among the berries (Table A1, Appendix A) [59,62,63,64,65,66,67,68,69,70,71,72,73,74,75,76]. In various studies on goji berries, variable values have been obtained for different assays, such as total polyphenol content (TPC), total flavonoid content (TFC), and total carotenoid content (TCC). These variations are primarily attributable to post-harvest techniques, extraction solvents, and the geographical origin of the samples [31,77,78,79].

For example, according to a study conducted by Mocan et al., Italian crops are richer in terms of phenolic compounds compared to Romanian crops, regardless of the mixing treatment. Polish crops, on the other hand, showed a high carotenoid content [64].

Additionally, a study conducted by Islam et al. analyzed goji berries collected in China, using a mixture of acetone/water/acetic acid for extraction. The TPC was reported at 31.6 mg GAE/100 g dw, while the TFC was 28.3 mg CAE/100 g dw. Substantially lower values were reported by Magalhães et al., who lyophilized the samples and extracted them with methanol, presenting significant differences that could be attributed to different methodologies [31,79].

Concerning post-harvest, over recent years, traditional methods like cold storage and treatments involving air and chemical substances have been commonly used to preserve goji berries. However, more recently, novel preservation techniques have emerged, showing promise in extending the storage duration and enhancing the shelf life of fresh goji berries, especially when implemented on an industrial scale. For instance, the application of hydrogen sulfide (H_2_S) effectively slowed down various aspects of deterioration in goji berries throughout their post-harvest storage. This included delaying decay, maintaining firmness, preserving color, flavor, total sugars and proteins, betaine, and ascorbic acid content. Moreover, H_2_S notably reduced the accumulation of substances like malondialdehyde (MDA), hydrogen peroxide (H_2_O_2_), and superoxide radicals (O_2_^−^). Additionally, it was observed that H_2_S boosted the activity of several important enzymes, such as catalase (CAT), ascorbate peroxidase (APX), peroxidase (POD), glutathione reductase (GR), and superoxide dismutase (SOD), while reducing the levels of 5-lipoxygenase (5-LOX) [80].

It has been observed that during the maturation of the fruits, some phenolic compounds accumulate, while others degrade. Phenolic compounds are intermediates in the phenylpropanoid metabolic pathway. The synthesis of these compounds changes based on alterations in enzymatic activity, whose change depends on the growth and maturation of the plant. Therefore, the various trends in phenolic compound content during maturation are likely due to different enzymatic activities involved in the biosynthetic pathways of these compounds [58,81].

These results emphasize the need to carefully consider study methodologies and geographic variables in the assessment of bioactive compounds in goji berries.

Phenolic components are reported as the main constituents of the antioxidant activity of *L. barbarum*; indeed, according to Chen et al., the increase in polyphenol content amplifies the DPPH scavenging capacity [82]. The antioxidant activity of *L. barbarum* fruits varies depending on the contribution of each polyphenol, thus making the fruit’s overall activity contingent upon its phenolic profile. Chlorogenic acid is one of the predominant phenolic acids in *L. barbarum* berries, known for preserving the integrity of the cell membrane and reducing the expression of inflammatory cytokines by inhibiting the NF-κB signaling pathway [59,83]. In addition to chlorogenic acid, polyphenols such as rutin, o-coumaric acid, caffeic acid, ferulic acid, and gentisic acid exert their antioxidant action by preventing excessive adaptive immunity and inflammation related to the endoplasmic reticulum (ER) stress, characterized by an accumulation of unfolded proteins in the ER, triggering activation of the UPR [84]. In particular, polyphenols can act on ER stress through the inhibition of the inositol-requiring enzyme 1α/X-box-binding protein 1 (IRE1α/XBP1) axis [85].

Additionally, naringenin, a flavanone found in goji extracts, exhibits anti-inflammatory capabilities by suppressing the secretion of tumor necrosis factor alpha (TNF-α), and some pro-inflammatory interleukins, such as IL-6 and IL-8.

Another consistent component in these berries is quercetin, a flavonol associated with the reduction of reactive oxygen species (ROS) induced by H_2_O_2_. Furthermore, according to Sharma et al., rutin appears to influence the p38/MAPKAPK2 and PI3K/Akt/GSK3β/NF-κB pathways in a murine model of dextran sulfate sodium (DSS)-induced chronic colitis [59,86,87].

The antioxidant activity of polyphenols is well known and results from a combination of their Fe ions chelating and oxidation-inhibiting properties, together with their inhibitory activity on oxidase enzymes widely involved in inflammatory diseases: 5-LOX, cyclooxygenase, soluble epoxide hydrolase, myeloperoxidase, NADPH oxidase, iNOS and xanthine oxidase [88,89,90,91,92].

In the COX-2 peroxidase assay, a potent inhibitory activity was observed in extracts derived from *L. ruthenicum* berries when compared to those derived from *L. barbarum*. Moreover, the expression levels of the pro-inflammatory cytokines’ genes IL-1β, IL-6, and TNF and the enzyme iNOS were evaluated upon pre-incubation of BV2 microglia cells [79].

**Figure 1:**
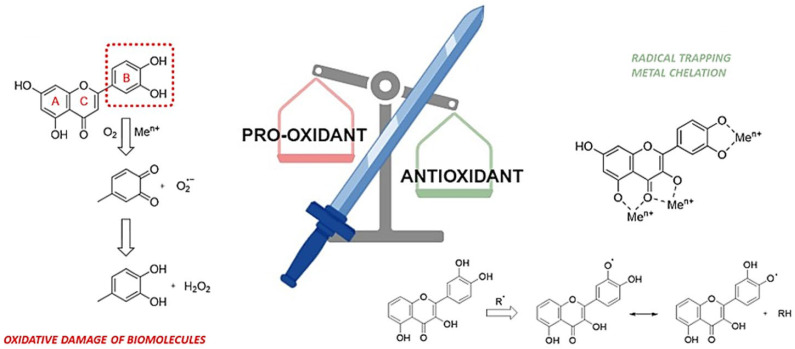
Polyphenols as a double-edged sword: some pro-oxidant and antioxidant mechanisms.

More controversial and less known, however, is their pro-oxidant capacity. This dual mechanism of polyphenols can be exploited for certain types of diseases, such as cancer, where the tumor microenvironment differs from that of healthy tissue in terms of ROS levels and metal ions (Figure 1).

Elevated levels of reactive oxygen species, such as H_2_O_2_, superoxide anion (O2•−), peroxynitrite (ONOO•−), and hydroxyl radical (OH•) can modify cellular biomolecules, inducing lipid peroxidation or DNA oxidation. In these mechanisms, ROS play a key role in the initiation and progression of carcinogenesis. In fact, tumor cells exhibit high levels of ROS due to increased cellular metabolism, alterations in O_2_ metabolism from oxidative phosphorylation, and the activity of NADPH oxidase. Elevated cellular ROS levels are associated with key aspects of carcinogenesis, including the induction of genetic alterations, cell proliferation, resistance to apoptosis, metastasis, and angiogenesis [93,94].

Flavonoids exhibit high free radical scavenging and chelating activities, and they are recognized as inducers of Nrf2 activity. This induction leads to the increased expression of detoxifying enzymes such as glutathione peroxidase (GPx) and UDP-glucuronosyltransferase (UGT). These enzymes form the primary enzymatic defense against electrophilic toxic substances.

Under certain conditions, such as high concentrations, elevated pH, and the presence of redox-active transition metals, phenolic compounds can act as pro-oxidants. The pro-oxidant activity of flavonoids involves their oxidation into o- or p-quinones, highly reactive towards nucleophilic thiols, amino acid groups in proteins, and glutathione. Alternatively, they may form labile redox complexes with metal cations. The B-ring structure of flavonoids, typically a catechol or a simple phenol, readily oxidizes, leading to the formation of electrophilic o-quinones. The latter can contribute to oxygen reduction, generating superoxide anions, and may also form adducts with DNA. Furthermore, besides the direct generation of ROS, polyphenol-induced changes in the redox milieu are influenced by molecules such as caffeic and chlorogenic acid. These compounds are known to elicit a pro-oxidant effect by stimulating intracellular ROS production via NADPH oxidase activation [95,96].

The pro-oxidant activity of polyphenols may also be mediated by transition metals, found at elevated levels in tumor cells, through the reduction in metal ions involved in redox cycles, promoting hydroxyl radical generation through the Fenton reaction. Consistent with this hypothesis, phenolic acids, including caffeic, chlorogenic, and ferulic acids, have been effective in inducing DNA cleavage in human promyelocytic leukemia cells (HL-60) in the presence of Cu(II) ions [97,98].

### 3.2. Carotenoid Compounds

Another crucial set of metabolites in wolfberries comprises carotenoids, whose content increases during the maturation process and are also responsible for the wolfberry’s red color. Zeaxanthin dipalmitate (ZD) is the predominant constituent, accounting for over 50% of the total carotenoids in the fruit. Additionally, zeaxanthin monopalmitate and a small amount of free zeaxanthin are also present. Recently, one study identified ZD and its two geometric isomers as fat-soluble constituents of the berries: 13Z-zeaxanthin dipalmitate and 9Z-zeaxanthin dipalmitate [44,99,100,101,102].

ZD is characterized by antioxidant, anti-inflammatory, and anti-apoptotic capabilities, primarily contributing to its hepatoprotective role in non-alcoholic fatty liver disease (NAFLD). In fact, in a non-alcoholic steatohepatitis (NASH) model, a malignant progression of NAFLD, and chronic hepatitis B (HBV), wild-type and HBV transgenic mice were treated with 2 mg/kg of ZD three times a week for eight weeks. The latter reduced steatosis and inflammation in diseased mice, thanks mainly to regulating the gene expression of antioxidant enzymes CAT and SOD-1 and lowered the activity of oxidative stress biomarkers 3-nitrotyrosine (3-NTR) and MDA. ZD treatment lowered all pro-inflammatory cytokines and chemokines such as TNF-α, IL-1ß, IL-6, and MCP-1, and reduced the activities of caspases 3/7 and 8 [103,104,105].

### 3.3. Polysaccharide Components

Polysaccharides isolated from *L. barbarum* fruits (LBPs) constitute a significant part of the extracts and are responsible for many biological activities attributed to them, especially antitumor, immunomodulatory, and neuroprotective activities.

LBPs are water-soluble glycoconjugates with a molecular weight ranging from 10 to 2300 kDa and account for approximately 5–8% of the dried fruits [46].

The polysaccharides of *L. barbarum* are mainly composed of (1→3)-β-D-galactopyranosyl, (1→6)-β-D-galactopyranosyl, and (1→4)-α-D-galactopyranosyl residues. Among the various constituents, a group of LBPs with a glycan-O-Ser structure has been considered for the efficacy of *L. barbarum*. Most LBPs are considered complex glycoproteins with differences in their composition, but although their composition may vary, most of their monosaccharides are the same: glucose, arabinose, galactose, mannose, rhamnose, and xylose [106,107,108].

*L. barbarum* polysaccharides are primarily isolated and purified from aqueous extracts of *L. barbarum*, yielding various polysaccharide fractions with different molecular weights (MWs). The relationship between MW and the intensity of contained polysaccharides has led to the identification of the most active portions: high-MW polysaccharides may scavenge free DPPH radicals and possess significant reduction capacity, while low-MW polysaccharides exhibit relatively stronger free radical scavenging activity, especially against hydroxyl radicals [109].

Consistent with this, according to Liu et al., the polysaccharide portion of *L. barbarum* has been purified into four fractions: LBP1 (225.6 kDa), LBP2 (140.2 kDa), LBP3 (645.0 kDa), and LBP4 (38.3 kDa). It has been specifically confirmed that the LBP4 fraction exerts notable antioxidant effects against superoxide anion radicals and exhibits an iron-reducing antioxidant effect in vitro. In general, albeit in different ways, all polysaccharides reduced levels of superoxide anions in mitochondria but failed to eliminate cytoplasmic H_2_O_2_, indicating that the antioxidant capacity of *L. barbarum* polysaccharides is achieved through the removal of superoxide anions from intracellular mitochondria [108,110].

It has been reported that the antitumor activity of LBPs is associated with fractions of high molecular weight. In fact, according to an in vivo study on H22 tumor-bearing mice, the fraction with medium molecular weight (40–350 kDa) was found to be active, capable of reducing tumor growth by around 40% [110,111].

The antioxidant function of LBPs is reflected in a significant improvement in parameters such as macrophage NO, phagocytic capacity, and acid phosphatase activity, demonstrating its immunomodulatory efficacy. Since macrophages play a broad and complex immune function, both in phagocytizing antigenic microorganisms and releasing cytotoxic molecules, they are generally an ideal model cell for evaluating immunological activity. It has been observed that polysaccharide-rich fractions, primarily consisting of arabinose and galactose, display enhanced activity. This is attributed to their ability to stimulate toll-like receptor 4 (TLR4) on macrophage surfaces, possibly influenced by their molecular weight. In fact, polysaccharide TLR4 ligands are much more active at molecular weights of 10–1000 kDa, and higher molecular weight polysaccharide fractions may have better immunological activity [112,113].

Under conditions of oxidative stress, LBPs increase cell viability, counteracting caspase-3 activation and ROS levels, significantly increase SOD and GSH-Px levels, and decrease MDA, TNF-α, IL-4, IL-6, MCP-1, and IL-17A content. These protective effects have been analyzed in mice and human pulmonary microvascular endothelial cells (HPMEC) induced by lipopolysaccharides (LPS). LBPs significantly attenuated LPS-induced lung inflammation and pulmonary edema in vivo, and restored LPS-stimulated endothelial cell (EC) migration dysfunction. Moreover, they also significantly suppressed NF-κB activation in vitro and reversed the release of cytochrome c. These results indicate that the anti-apoptotic and antioxidant properties of LBP could partially protect against acute respiratory distress syndrome (ARDS) [114].

Furthermore, LBPs are also known to protect against neurotoxicity by upregulating the Nrf2-HO-1 pathway in cells against alcohol-induced oxidative damage [115].

### 3.4. Melatonin

Melatonin, a hormone primarily associated with regulating sleep–wake cycles, has been identified in various plant sources, including goji berries. Studies have shown that goji berries contain significant levels of melatonin, although the exact concentration may vary depending on factors such as growing conditions, maturity at harvest, and post-harvest handling [116].

The presence of melatonin in goji berries is of interest due to its potential health benefits. Melatonin’s antioxidant properties, as well as its pivotal role in various anticancer mechanisms, encompass apoptosis induction, inhibition of cell proliferation, reduction in tumor growth and metastases, alleviation of chemotherapy and radiotherapy side effects, attenuation of drug resistance in cancer therapy, and enhancement of the therapeutic efficacy of conventional anticancer treatments [117].

While goji berries are recognized as a natural source of melatonin, further studies are needed to fully understand the bioavailability and health implications of melatonin derived from dietary sources, including its potential synergistic effects with other bioactive compounds present in goji berries.

## 4. Antitumor Activity of *Lycium barbarum*

### 4.1. Main Antitumor Activity of Single Components: An Overview

#### 4.1.1. Zeaxanthin

As stated in Section 3.2, carotenoids represent a crucial component of *L. barbarum* extracts. In particular, zeaxanthin has demonstrated potential effects against breast cancer, gastric cancer, and melanoma due to its dual pro-oxidant/antioxidant role explained above. Specifically, according to Sheng et al., this carotenoid has the capability to increase intracellular ROS production in gastric adenocarcinoma (AGS) cells. This leads, on one hand, to an alteration in the protein kinase B (PKB), also known as Akt and Signal transducer and activator of transcription 3 (STAT3) signaling pathways, resulting in reduced expression of cyclin B1 and A, inducing G2/M cell cycle arrest. On the other hand, these pathways also regulate MAPK, Nf-κB signaling, leading to an increase in caspase-3 and Poly ADP-ribose polymerase (PARP). Both pathways lead to cell apoptosis [118,119,120].

In vitro studies on malignant melanoma cells A375 have shown that zeaxanthin-rich extracts selectively influence extracellular signal-regulated kinase (ERK), c-Jun N-terminal kinase (JNK), and p38 compared to human fibroblast cell line BJ [121]. These effects could always be associated with the pro-oxidant action shown by these compounds, by activating MAPKs, which are sensitive to redox balance. Moreover, this would activate a vicious cycle since the increased phosphorylation expression of JNK, ERK1/2, or p38 may lead to a pro-oxidative process within tumor cells, detrimental to tumor cell survival. Moreover, zeaxanthin significantly downregulates the expression of membrane CD44 and CD105 in A375, resulting in reduced endothelial cell migration and cell adhesion. Both CD44 and CD105 have been identified as potential biomarkers for cancer prognosis, since CD44 is associated with cancer stem cells and acts as a receptor for hyaluronic acid, facilitating cell adhesion to the extracellular matrix, while CD105 is a cell surface receptor for transforming growth factor-beta (TGF-β) involved in angiogenesis [122,123].

These findings suggest the applications of zeaxanthin, derived from *L. barbarum* extracts, as a cytoprotective agent in tumor models and as an anticancer prodrug in combination with standard therapy.

#### 4.1.2. Polyphenol Fraction

Regarding polyphenols, although they exert a strong impact on oxidative stress in cells, there are fewer studies demonstrating their anti-proliferative efficacy against cancer cells compared to other similarly abundant components in goji berries, such as polysaccharides. Several research articles have documented their antitumor efficacy, notably against breast cancer cells (Section 4.2). However, the precise mechanism remains incompletely understood, and specific components responsible for the antitumor activity have not been conclusively identified. Hence, further investigations are warranted to elucidate these aspects. In addition, some work reports that the polyphenolic component has an indirect anticancer action by stimulating immune cells. For instance, according to Kwaśnik et al., extracts from goji berries significantly enhanced the proliferation of natural killer cells (NK-92), which constitute the body’s first line of defense against cancer. Unlike other extracts, such as chlorella, goji berry extracts have demonstrated a substantial reduction in lactate dehydrogenase (LDH) levels, without impacting the integrity of NK cell membranes. The enhancement of immunomodulatory properties was tested in co-culture with the human colon cancer cell line LS180, leading to an almost 100% reduction in cell tumor vitality [124].

#### 4.1.3. Polysaccharide Fraction

Many studies have indicated that LBPs also exert their effects on tissues or tumor cells. For instance, exposure to aqueous glycopeptide fraction (LBGP) rich in arabinogalactan derived from *L. barbarum* LBPs has particularly shown stronger inhibitory effects on cervical cancer cells (HeLa), gastric carcinoma cells (SGC-7901), and human breast cancer cells (MCF-7). The investigation into the underlying mechanism suggested that the extracts inhibited tumor cell growth by arresting the cell cycle in the G0/G1 phase, disrupting mitochondrial function, inducing oxidative stress, and regulating the MAPKs pathway, thereby inducing apoptosis without inducing toxicity to normal cells in vitro [125]. Once more, these findings align with the concept of a dual mechanism wherein natural compounds act as antioxidants in healthy cellular environments rather than pro-oxidants in tumor environments because of the different redox environment in cellular compartments.

In colorectal cancer, the use of LBPs has demonstrated a decrease in the cellular vitality of SW480 and Caco-2 cells, showing a dose-dependent arrest in the G0/G1 phase of the cell cycle. Experiments on glioma animal models revealed that LBP can suppress tumor growth and promote the invasion of CD8+ T cells into the brain, indicating a potential role in regulating the blood–brain barrier. Additionally, investigations on human hepatoma cells (QGY7703) highlighted that LBPs inhibit cell growth, induce cell cycle arrest in the S phase, and stimulate apoptosis, primarily through an increase in intracellular calcium in the apoptotic process [126].

The effects of LBPs on the growth of human prostate cancer cells were examined in vitro and in vivo by Luo et al. LBPs inhibited the growth of PC-3 and DU-145 cells in a dose- and time-dependent manner by reducing the ratio of Bcl-2/Bax expression following LBPs treatment. This led to the inhibition of proliferation and induction of apoptosis. The animal study demonstrated that LBP significantly inhibited the growth of PC-3 xenografts in nude mice, with a substantial reduction in tumor volume and weight in the LBP-treated group compared to the control group [127].

The antitumor efficacy of LBPs derives mainly from the inhibition of cell growth, cell cycle arrest, and induction of apoptosis, according to various mechanisms.

The phosphoinositide 3-kinases (PI3K) and their downstream mediators Akt and mTOR constitute the PI3K/Akt/mTOR signaling cascade, which regulates abnormal cell proliferation and differentiation and promotes tumor cell growth. This signaling pathway is involved in the cellular response to extracellular stimuli, including insulin-like growth factor 1 (IGF-I), epidermal growth factor (EGF), and fibroblast growth factor (FGF).

LBPs may play essential roles in the antitumor action by regulating the PI3Ks-Akt-mTOR signaling pathway. Phosphorylation of Akt generates p-Akt, which can further activate the mTOR pathway, increasing its expression, and enhances the proliferation and migration of tumor cells.

Conversely, LBPs can reduce the expression of p-Akt, inhibiting tumor cell proliferation and migration [128,129].

Various in vitro studies have indicated that LBPs can safeguard cell damage by promoting cellular autophagy through the activation of p38-MAPK and the expression of ERK. Simultaneously, they can prevent apoptosis by activating the expression of ERK and p53, thereby exerting an anticancer effect [130].

Dendritic cell (DC) maturation is critical for the initiation of the immune response. Activated DCs enhance T cell targeting through the TLR4 signaling pathway. Here, phosphorylation of ERK, JNK, p38 mitogen-activated protein kinase, and Nf-κB are the molecules in the valley of TLR4. *L. barbarum* polysaccharides have been reported to induce TLR4-mediated functional activation of DCs through NF-κB activation [131].

### 4.2. Lycium barbarum and Breast Cancer

Breast cancer is the most common cancer in women and the second leading cause of cancer-related death in women worldwide. Recently, there has been a growing interest in the antitumor action of *L. barbarum* on breast cancer, and the principal results obtained so far are reported below.

The polyphenolic fraction of *L. barbarum* has been demonstrated to have significant antioxidant effects, which are correlated with its cytotoxicity against breast cancer cell lines. On the other hand, the polysaccharide fraction contributes to the antiproliferative effects of *L. barbarum* [132].

A study evaluated the proliferation, apoptotic, and necrotic effects of different concentrations of an ethanol extract of goji berries, containing a higher percentage of polyphenols, against the invasive ductal carcinoma T-47D cell line. The results demonstrated a significant decrease in cell proliferation after exposure to the extract in a time-dependent manner, up to 96 h. Analyses highlighted a disturbance in the cell cycle, with a significant increase in the expression of the proteins p21 and p53 and a slight decrease in the expression of CDK6 and cyclin D1. However, the antitumor activity of the goji berry extract is mainly attributed to apoptotic effects through the mitochondrial pathway, as demonstrated by a significant dose-dependent increase in the pro-apoptotic protein Bax expression and a decrease in the expression of the anti-apoptotic protein BclxL after treatment in T-47D cells [133].

In triple negative MDA-MB-231 cells, *L. barbarum* extracts inhibit the phosphorylation of the epidermal growth factor receptor (EGFR), regulated by ERK. This study demonstrates that the phosphatidylinositol-4,5-bisphosphate 3-kinase (PI3K)/Akt signaling pathway is involved in the induction of cell death induced by goji berry extract. Furthermore, the treatment inhibited the expression of anti-apoptotic Bcl-2 and enhanced the pro-apoptotic expression of Bax at transcriptional levels, inducing apoptosis in tumor cells through the activation of caspase-9 and caspase-3. Activation of these apoptotic caspases was also detected in MCF-7 cells after treatment with the LBGP fraction, along with a decrease in the Bcl-2/Bax ratio and mitochondrial membrane potential. A significant decrease in T-SOD and CAT activities, as well as GSH-Px activity and GSH content, was also observed [125,134].

The alteration of the ERK pathway is complex because it has been demonstrated that both the activation and inhibition of this pathway induce cell growth, differentiation, or apoptosis in breast cancer. In fact, in contrast to the previous study, Shen et al. demonstrated that on MCF-7 cells, LBPs increased ERK activity in a dose-dependent manner. Consequently, they caused the activation of p53, an upstream regulator of ERK activation, leading to apoptosis in breast cancer cells [135,136].

Recently, it has been demonstrated that certain aqueous extracts containing LBPs inhibit the in vitro growth of the ER+ human breast cancer MCF-7 cell line, suggesting the alteration of estradiol cellular metabolism as a mechanism [137].

The growth-inhibitory effects of LBPs were examined in a dose-dependent manner over 7 days. This demonstrated that LBPs downregulate tumor growth promoting the increase of the 2-hydroxylation pathway with the production of antiproliferative metabolites of E2 (estradiol, strongly mitogenic) and/or facilitate the transformation of 16α-OHE1 (estrone, mildly mitogenic) into E3 (estriol, antimitogenic).

Considering the close interaction between estradiol and IGF-I, a growth/angiogenic factor whose action is mediated by the IGF-I receptor (IGF-1R), in the regulation of mammary epithelium and breast cancer cell growth, connections between the antitumor effects of LBPs and IGF-I-mediated signal transduction pathways have been observed [138].

The ER+ MCF-7 breast cancer cell line shows a strong growth response to IGF-I, while the ER- MDA-MB-468 breast cancer cell line does not respond to IGF-I-induced growth stimulation. LBPs have been found to inhibit IGF-I-stimulated proliferation of MCF-7 cells in a dose- and time-dependent manner. The treatment reduced IGF-I protein levels, indicating that these compounds from the berries exhibit strong anti-angiogenic effects. Like many natural substances, LBPs also exert anti-angiogenic effects, but the underlying molecular mechanisms remain largely unknown [138,139].

Huang et al. demonstrated that LBPs significantly inhibited the autocrine IGF-I-induced expression of VEGF mRNA and protein secretion, indicating that LBPs influence VEGF expression in MCF-7 cells. This inhibition of angiogenesis is partly attributed to blocking the accumulation of hypoxia-inducible factor HIF-1α, but not its mRNA, caused by decreased levels of PI3K protein and PI3K phosphorylation [139].

LBPs are also responsible for an atypical form of cell death in MCF-7 and MDA-MB-231 cell lines, termed ferroptosis.

Ferroptosis is a non-canonical cell death that differs from apoptosis and autophagy. Various natural compounds induce ferroptosis in different in vitro and in vivo cancer models. Ferroptosis induces cell demise via the iron-driven buildup of lipid peroxides, which produce ROS through the Fenton reaction. These reactive species then attack the polyunsaturated fats in cell membranes, initiating lipid peroxidation and ultimately leading to cell death. An important regulator of ferroptosis is the micronutrient selenium, necessary for the biosynthesis of selenoproteins that eliminate ROS, including a key inhibitor of phospholipid peroxidation, GPX4. Cystine, in its oxidized form, also opposes ferroptosis by contributing to GPX4 activity [140,141].

Many tumor cells exhibit increased susceptibility to ferroptosis, and the induction of ferroptosis could be explored as an antitumor therapy. Moreover, considering the iron-chelating properties commonly attributed to polyphenols and polysaccharides previously described, along with their capacity to function as a Trojan horse in pro-oxidant environments, they emerge as potent inducers of ferroptosis. These attributes may provide insight into the observed mechanism of action of *Lycium barbarum*.

While several studies have explored the antitumor effects of LBPs, the mechanisms of tumor cell death induced by LBPs have not been fully elucidated. According to Du et al., LBPs inhibited cell proliferation and arrested the cell cycle, causing changes in the expression levels of Cyclin E and CDK2 proteins in MCF-7 and MDA-MB-231. Furthermore, LBPs induced lipid peroxidation and released an excess pool of free iron, known as a labile iron pool (LIP). A LIP can promote the formation of lipid ROS through the Fenton reaction, leading to ferroptosis. Additionally, the results showed that LBP-induced ferroptosis in breast cancer cells was caused by the downregulation of the expression of the light chain subunit of the cystine/glutamate antiporter system (xCT) and GPX4, which play a vital role in antagonizing ferroptosis by regulating GSH synthesis. GPX4 inactivity and the repression of SLC7A11 (the gene for xCT) lead to ROS accumulation, thereby modulating ferroptosis [135,142,143,144].

As evidence that extracts from the fruits of *L. barbarum* can be potential partners in the combined treatment of breast cancer, several combination tests were conducted with other well-known drugs, such as doxorubicin (DOX), an anthracycline antineoplastic agent. On one hand, an increase in antitumor activity was observed, and on the other hand, a potential dose-dependent reduction in the risk of cardiotoxicity in the anthracycline therapeutic regimen against breast cancer was observed [145,146].

Anthracycline antibiotics are among the most potent and commonly used chemotherapeutic agents. Nevertheless, their cardiotoxicity and nephrotoxicity represent the primary limiting factors, yet the precise mechanisms underlying organ toxicity remain incompletely elucidated. In this regard, there are two main theories: the formation of iron-bound free radicals and the formation of the doxorubicinol metabolite, inducing mitochondrial dysfunction with subsequent activation of apoptotic and necrotic processes. Furthermore, due to relatively lower levels of CAT and GSH-peroxidase in cardiomyocytes, the heart is more susceptible to oxidative damage compared to other tissues. Therefore, substances that reduce oxidative stress and stabilize mitochondrial dysfunction would alleviate damage to the heart and renal tissue caused by anthracyclines, especially DOX [147,148,149].

Georgiev *et al*.’s studies have demonstrated that after co-administration of DOX and an extract of *L. barbarum*, there were synergistic effects at low concentrations of doxorubicin (0.02–0.075 µM) and additive effects with increasing concentrations of DOX on MCF-7 cells. Only at the highest concentration of doxorubicin (0.6 µM) with the extract is an antagonistic action rather than additive evaluated.

In MDA-MB-231 cells, low concentrations of doxorubicin (0.02–0.075 µM) with the extract showed more antagonistic effects than additive, while at high concentrations (0.15–0.6 µM), the observed combination responses were synergistic [132,150].

Furthermore, studies conducted on male Sprague Dawley rats have demonstrated that the polysaccharide fractions of *L. barbarum* can reduce doxorubicin-induced cardiotoxicity, suggesting a cardioprotective effect against DOX-related oxidative stress. Pretreatment with LBPs significantly prevented the loss of myofibrils, reduced arrhythmias, and improved the cardiac function of rats treated with DOX, as evidenced by lower mortality (13%), better antioxidant activity, and biochemical cardiac markers, such as aspartate aminotransferase (AST) and serum creatine kinase (CK) levels [151].

In addition, beyond the antitumor activity, the immunoprotective action of a water-soluble fraction of LBPs has also been evaluated. This fraction can mitigate the typical immunotoxicity caused by DOX in mice. Indeed, the results have shown that it alleviated the immunosuppression induced by DOX by promoting the recovery of the cell cycle of bone marrow cells (BMC) and improving the peripheral blood lymphocyte count.

It is known that the bone marrow is the organ most affected during any immunosuppressive therapy, and the suppression of BMC would hinder the regeneration of new blood cells, resulting in thrombocytopenia and leukopenia, which could lead to significant morbidity and mortality [146,152].

Other fractions of *L. barbarum* were also examined to trace whether all components are responsible for the observed effects with doxorubicin. It was found that the use of a fraction devoid of polysaccharides/rich in polyphenols with DOX on mature male albino Wistar rats also led to a significant reduction in DOX IC_50_. Similarly, after the analysis of cardiotoxicity markers, an improvement in cardiac function compared to rats that did not receive the extract administration was observed.

This highlights that the organ-protective effect is primarily attributed to the antioxidant capacity typical of all the ingredients in goji berries [153].

Further, Sun et al. aimed to enhance *L. barbarum*’s anticancer impact by integrating its extract into photothermal therapy, known for minimal side effects and targeted treatment outcomes. They utilized polypyrrole nanoparticles (PPy NPs) as a promising photothermal agent triggered by near-infrared (NIR) light [154]. This multifaceted approach sought to improve the effectiveness of localized doxorubicin treatment for breast cancer, aligning with emerging possibilities in clinical nanomedicine applications. The study concludes that combining DOX, LBP, and Ppy NPs for localized breast cancer treatment in a tumor-bearing (4T1) Balb/c mouse model, along with photothermal therapy, offers a promising strategy. By reducing DOX dosage and systemic distribution, it not only enhances DOX’s antitumor impact but also mitigates systemic toxicity and improves the anti-inflammatory immune response, showing reduced IL-10, IgA, and ROS levels alongside increased IFN-γ and TNF-α levels, indicating its potential for future clinical cancer treatments [155].

Finally, to our knowledge, no clinical trials employing *L. barbarum* as a potential direct anticancer agent are reported; however, for completeness, we report the only clinical trials in reference to *L. barbarum* where it is used as an anticancer adjuvant because of its ability to stimulate an immune response. In particular, Cao et al. [156] conducted a clinical investigation to evaluate the impact of co-administration of a lymphokine-activated killer polysaccharide (LAK)/IL-2 extract and LBPs on patients with advanced cancer, including breast cancer patients (comprising 79 individuals). Their results revealed that patients treated with a combination of LAK/IL-2 and LBP showed a higher response rate and more prolonged mean tumor regression than those given LAK/IL-2 alone. In addition, therapy combining LAK/IL-2 with LBP induced a more substantial increase in NK and LAK activity than LAK/IL-2 monotherapy.

**Figure 2:**
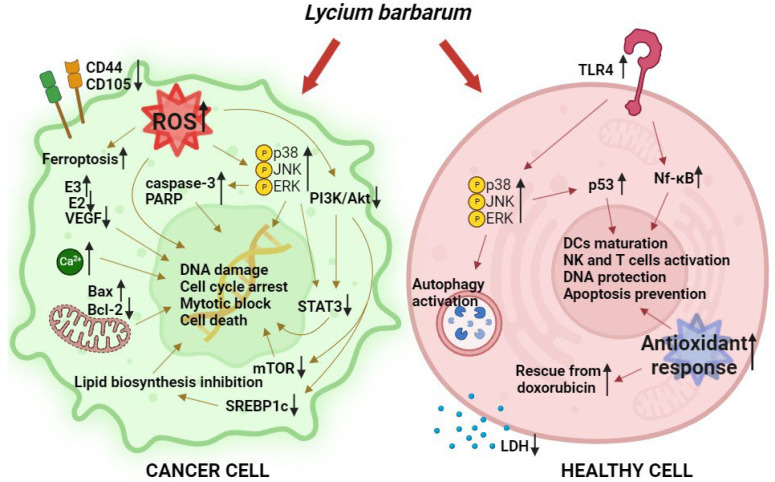
Main activated biochemical pathways following *L. barbarum* administration in cancer and healthy cells.

In summary, Figure 2 encapsulates the primary toxicity mechanisms outlined for tumor cells and the protective mechanism activated in healthy compartments discussed throughout this report. We believe that this summary of the current state of knowledge regarding the anticancer properties of *Lycium barbarum* could be valuable, considering the necessity for a thorough exploration of the individual components of bioactive goji berries that contribute to their health-promoting qualities. This need arises from the need to establish a clear cause-and-effect association between goji berry consumption and health outcomes, which can only be achieved through accurate characterization and standardization of goji berry composition, which unfortunately is not present in much of the reported works. In addition, a more thorough investigation of the mechanism could be warranted to assess the impact of integrating these beneficial phytochemical compounds.

In this regard, network pharmacology has emerged as an innovative approach for studying the mechanisms of herbal medicine. It involves screening active ingredients of natural drugs and exploring multiple components of drugs, targets of action, and potential mechanisms of action on diseases. Hu et al. utilized these techniques to investigate the therapeutic effects of wolfberry on breast cancer. They screened active components and their targets, constructed ingredient–target–disease and protein–protein interaction networks, and performed GO (Gene Ontology) and KEGG (Kyoto Encyclopedia of Genes and Genomes) enrichment analyses [157].

Their findings provided a scientific basis for wolfberry’s clinical application in treating breast cancer and developing antitumor drugs. Core targets identified in the protein–protein interaction (PPI) network included ESR1 (Estrogen Receptor 1), MYC (Myelocytomatosis oncogene), HIF1A (Hypoxia Inducible Factor 1 Alpha), EGFR (Epidermal Growth Factor Receptor), VEGFA (Vascular Endothelial Growth Factor A), and CCND1 (Cyclin D1), representing key molecular pathways in breast cancer development and progression. Understanding the roles of these targets is crucial for developing targeted therapies and identifying novel biomarkers for breast cancer diagnosis and prognosis [158].

Additionally, GO enrichment analysis revealed that wolfberry’s main biological processes against breast cancer include responses to steroid hormones, ketones, and chemical stress, as well as epithelial cell proliferation. KEGG enrichment analysis indicated that wolfberry’s anti-breast cancer targets are primarily enriched in cancer pathways, the estrogen signaling pathway, AGE-RAGE signaling pathway, P53 signaling pathway, and HIF-1 signaling pathway. These insights contribute to understanding wolfberry’s potential as a therapeutic agent for breast cancer treatment.

## 5. Conclusions

Goji berries exhibit nutraceutical properties due to their rich phytochemical composition, which includes polyphenols, flavonoids, organic acids, carotenoids, polysaccharides, glycopeptides, and vitamins. These compounds underlie the berries’ remarkable biological activities, such as antioxidant, antitumor, antimicrobial, hypoglycemic, hypolipidemic, immunomodulatory, and prebiotic activities.

In particular, *L. barbarum* extracts have been associated with several mechanisms that may contribute to inhibition of tumor growth and promotion of cell death in tumors.

First, the antioxidant properties of the polyphenols present in goji berries have been linked to their ability to counteract oxidative stress, a factor that can promote tumor development. In addition, antiproliferative effects have also been observed in various cancer cell lines, including breast cancer, highlighting the potential ability to limit the growth and division of cancer cells.

Studies have indicated that the polysaccharides present in goji berries may also positively influence cell cycle regulation by inhibiting proliferation and inducing apoptosis in cancer cells. The anticancer action appears to involve key cell signaling pathways involved in cell growth and survival, including NK cells activation and bolstering their cytotoxic functions against cancer cells. These polysaccharides are believed to stimulate various immune responses, potentially aiding the body’s defense mechanisms against cancer.

Results of reported studies on the antitumor actions of *L. barbarum* suggest that its bioactive compounds could be used as part of adjuvant therapeutic approaches in the potential treatment of cancer, contributing to the chemoprevention and control of cancerous growth.

These show numerous advantages, foremost among them being selectivity towards tumor cells, having no cytotoxic effects on the corresponding healthy lines. This gives an important indication that the in vivo co-administration of known anticancer agents with *L. barbarum* reduces the numerous adverse effects.

Another positive factor would be the decrease in drug resistance, a strongly increasing problem in the treatment of chronic diseases, such as cancer. In fact, there is already a great deal of research on trying to find a solution by designing new molecules that can act on cellular resistance pathways, or using already known molecules that can act on multiple pathogenic targets. Therefore, *L. barbarum* seems to be able to perform this function as well, both because the components of the extract act on multiple molecular pathways, and the addition of these to therapies could lead to a decrease in the dosage of standard drugs.

For these reasons, it is important to continue research into the biochemical mechanisms underlying the antiproliferative effect on tumor cells.

Overall, the existing studies lay a foundation suggesting the potential anti-cancer properties of goji berries, but more robust clinical trials focusing specifically on their effects on breast cancer in humans are needed for clearer conclusions.

## Figures and Tables

**Table 1:** Some examples of natural substances and nutraceuticals widely studied for their beneficial properties.

N.	Phytochemicals	Bioactivity	Disease	Refs.
**1**	Dairy-derived β-lactoglobulin peptide	ROS reduction, Nrf2 activation and expression of cytoprotective enzymes such as NADPH oxidase.	Intestinal inflammation.	[11]
**2**	Chitosan	Reduction in total cholesterol, triglycerides, LDL, and VLDL.	Hyperlipidemias.	[8]
**3**	Glycyrrhizin	Alteration of intracellular transport of viral antigens and suppresses sialylation of the surface antigen (HBsAg) of hepatitis B virus (HBV).	Chronic hepatitis B.	[12,13]
**4**	Capsaicin	Selective activation of the Ca^2+^-permeable ion channel, TRPV1.	Acute pain, neuropathic pain, and inflammatory pain.	[14] https://www.sciencedirect.com/science/article/pii/S1756464620305648-b0580 (accessed on 22 February 2024)
**5**	Menthol	Agonism of the Ca^2+^-permeable ion channel TRPM8, and the k-opioid receptor, OPRK1.	Acute pain, neuropathic pain, and inflammatory pain.	[23,24]
**6**	Curcumin	Inhibition of MAPK and Nf-κB signaling pathways.	Inflammatory pathologies, cardiovascular disorders.	[15]
**7**	Catechins	PPAR-γ agonists. Powerful antioxidant effect due to the chelating capacity of metal ions.	Atherosclerosis, cardiac hypertrophy, neurodegenerative disorders.	[16,17]
**8**	Sulphur-containing compounds from onions and scallions	Stimulation of the production of detoxification enzymes, such as GSTs.	Hepatic dysfunction.	[18,20]
**9**	Coffee silver skin melanoidins	Reduction in intracellular ROS and inhibition of MMPs.	Skin aging and related diseases.	[25]
**10**	*Spirulina platensis*, *Moringa oleifera*, *Ganoderma lucidum* bioactive compounds	Reduction in NLRP3 expression and Nf-kB levels in the myocardium.	Heart failure and fibrosis.	[21,22]

## Data Availability

Not applicable.

## Appendix A

**Table A1:** Polyphenols found in *L. barbarum*.

N.	Phenolic Compounds	Classification	Refs.
**1**	Catechin	Flavan-3-ols	[63,64]
**2**	Epicatechin	Flavan-3-ols	[63,64]
**3**	Naringenin	Flavonones	[63,65]
**4**	Hesperidin	Flavonones	[63,65]
**5**	Apigenin	Flavones	[63,66]
**6**	Luteolin	Flavones	[63,66]
**7**	Derrone	Isoflavones	[63,67]
**8**	Alpinumisoflavone	Isoflavones	[63,67]
**9**	Auriculasin	Isoflavones	[63,67]
**10**	Kaempferol	Flavonols	[63,68]
**11**	Quercitin	Flavonols	[63,68]
**1** **2**	Myricetin	Flavonols	[63,68]
**13**	Rutin	Flavonols	[63,68]
**14**	Quercetin-rhamno-di-hexoside	Flavonols	[63,68]
**15**	7-*O-β*-D-Glucopyranosyl-rutin	Flavonols	[63,67]
**16**	Hyperoside	Flavonols	[63,66]
**17**	Morin	Flavonols	[63,66,69,70]
**18**	Nicotiflorin	Flavonols	[63,66,69,70]
**19**	Narcissin	Flavonols	[63,66,69,70]
**20**	Isoquercitrina	Flavonols	[63,66]
**21**	Proanthocyanidin-A2	Tannins	[63,65]
**22**	Proanthocyanidin-B2	Tannins	[63,65]
**2** **3**	Ellagic acid	Tannins	[63,65]
**24**	Gallic acid	Tannins	[63,65]
**2** **5**	Scopoletin	Coumarins	[63,67,69]
**26**	Fabiatrin	Coumarins	[63,67,69]
**27**	Scopolin	Coumarins	[63,67,69]
**28**	Esculetin	Coumarins	[62,67]
**2** **9**	Lycibarbarcoumarin A	Coumarins	[63,67,69]
**30**	Pinoresinol	Lignans	[63,67]
**31**	Medioresinol	Lignans	[63,67]
**32**	Syringaresinol	Lignans	[63,67]
**33**	4-*O*-(β-d-glucopyranosyl)syringaresinol	Lignans	[62,67]
**34**	Acanthoside B	Lignans	[63,67]
**35**	Arctigenin	Lignans	[63,67]
**36**	Arctiin	Lignans	[63,67]
**37**	Neolignan *threo*-1,2-bis(4-hydroxy-3-methoxyphenyl)-1,3-propanediol	Lignans	[63,67]
**38**	Neolignan *erythro*-1,2-bis(4-hydroxy-3-methoxyphenyl)-1,3-propanediol	Lignans	[63,67]
**39**	(*β*)-Lyoniresinol 3-O-*β*-d-glucopyranoside	Lignans	[62,67]
**40**	Medioresinol	Lignans	[62,67]
**41**	Cannabisin D	Lignanamides	[63]
**42**	Cannabisin E	Lignanamides	[63]
**43**	Cannabisin F	Lignanamides	[63]
**44**	*Threo*-Canabisine H	Lignanamides	[63]
**45**	*Erythro*-Canabisine H	Lignanamides	[63]
**46**	Melongenamide D	Lignanamides	[63]
**47**	Grossamide	Lignanamides	[63]
**4** **8**	Lyciumamide A	Lignanamides	[63,71]
**49**	Lyciumamide B	Lignanamides	[63,71]
**50**	Lyciumamide C	Lignanamides	[63,71]
**51**	*p*-Hydroxybenzoic acid	Hydroxybenzoic acids	[63,65,68,71,72]
**52**	Vanillic acid	Hydroxybenzoic acids	[63,65,68]
**5** **3**	2,4-Dihydroxybenzoic	Hydroxybenzoic acids	[63,65,68]
**5** **4**	Veratronic acid	Hydroxybenzoic acids	[63,65,68]
**55**	Benzoic acid	Hydroxybenzoic acids	[63,65,68]
**56**	Salicylic acid	Hydroxybenzoic acids	[63,65,68]
**57**	Syringic acid	Hydroxybenzoic acids	[63,65,68]
**58**	Chlorogenic acid	Hydroxycinnamic acids	[63,68,69,73,74]
**59**	Caffeic acid	Hydroxycinnamic acids	[63,68,69,73,74]
**60**	Coumaric acid	Hydroxycinnamic acids	[63,68,69,73,74]
**61**	Ferulic Acid	Hydroxycinnamic acids	[63,68,69,73,74]
**62**	Dicaffeoylquinic acid	Hydroxycinnamic acids	[63,74]
**63**	Methylchlorogenate	Hydroxycinnamic acid derivatives	[63,67,75]
**64**	Lycibarbarphenylpropanoid A	Phenylpropanoids	[63,67,75]
**65**	Lycibarbarphenylpropanoid B	Phenylpropanoids	[62,67]
**66**	Lycibarbarphenylpropanoid C	Phenylpropanoids	[62,67]
**67**	Lycibarbarphenylpropanoid D	Phenylpropanoids	[62,67]
**68**	Lycibarbarphenylpropanoid E	Phenylpropanoids	[62,67]
**69**	Lycibarbarphenylpropanoid F	Phenylpropanoids	[62,67]
**70**	Lycibarbarphenylpropanoid G	Phenylpropanoids	[62,67]
**71**	Lycibarbarphenylpropanoid H	Phenylpropanoids	[62,67]
**72**	Lycibarbarphenylpropanoid I	Phenylpropanoids	[62,67]
**73**	Lycibarbarphenylpropanoid M	Phenylpropanoids	[63,67,75]
**74**	Syringenin	Phenylpropanoids	[62,67]
**75**	Isoscopoletin	Phenylpropanoids	[62,67]
**76**	Ethyl-4-*O-β*-d-glucopyranosyl-E-ferulate	Phenylpropanoids	[62,67]
**77**	Phloretic acid	Phenolic acids	[63,67]
**78**	Dihydroferulic acid	Phenolic acids	[63,67]
**79**	Ethyl Dihydroferulate	Phenolic acid derivates	[63,67]
**80**	Arbutin	Phenolic acid derivates	[63,74]
**81**	*p*-Hydroxybenzaldehyde	Phenolic acid derivates	[63,67]
**82**	Protocatechuic aldehyde	Phenolic acid derivates	[63,73]
**83**	N-*Trans*-feruloyl tyramine	Phenolic acid amides	[63,73]
**84**	N-*Trans*-feruloyl 3-methoxyturamine	Phenolic acid amides	[63,73]
**85**	Lyciumide A	Phenolic acid amides	[63,71,76]
**86**	N-*Trans*-*p*-coumaroyl tyramine	Phenolic acid amides	[63,71,76]
**87**	N-*Cis*-*p*-coumaroyl tyramine	Phenolic acid amides	[63,71,76]
**88**	N-feruloyl agmatine	Phenolic acid amides	[63,71,76]
